# Patient expectations for outcome with psychological intervention for psychosis

**DOI:** 10.1016/j.schres.2026.03.005

**Published:** 2026-06

**Authors:** Daniel Freeman, Laina Rosebrock, Felicity Waite

**Affiliations:** a Department of Experimental Psychology, University of Oxford, Oxford, UK; b Oxford Health NHS Foundation Trust, Oxford, UK

**Keywords:** Psychological intervention, Credibility, Expectancy, Persecutory delusions, Outcomes

## Abstract

**Background:**

Initial patient perceptions of a treatment's credibility, and expectations for improvement, have been shown to predict to a small degree outcomes for common mental health disorders. This study aimed to discover: how patients with psychosis initially perceive psychological therapy's credibility and likely success; whether there are predictors of these first views; and, primarily, if such ideas predict improvement in persecutory delusions.

**Methods:**

We analysed first therapy session data on credibility and expectancy from 195 patients with non-affective psychosis taking part in clinical trials treating persecutory delusions. Baseline assessments before randomisation to therapy were used to identify potential predictors of credibility and expectancy. First session credibility and expectancy scores were tested as predictors of persecutory delusion severity six months later.

**Results:**

Most patients were optimistic about therapy's potential. Baseline delusion severity did not predict credibility, β = −0.02, *p* = .742, or expectancy, β = −0.03, *p* = .632. Higher psychological well-being predicted higher levels of expectancy, β = 0.23, *p* = .001. Higher levels of credibility, β = −0.17, *p* = .021, and expectancy, β = −0.17, *p* = .020, predicted lower severity of persecutory delusions six months later. Credibility, β = 0.00, *p* = .970, and expectancy, β = −0.21, *p* = .074, did not significantly predict uptake of therapy sessions.

**Conclusions:**

Treatment credibility and expectancy may account for a small proportion of improvement when psychological interventions are used to treat severe paranoia. The results are comparable to those for common mental health disorders. There was little prediction of patient variability in credibility and expectancy. It will be helpful to understand a patient's initial views on intervention so that any concerns can be addressed.

## 1 Introduction

Initial patient perceptions of a psychological treatment's credibility predict later outcomes. A meta-analysis with one and a half thousand patients showed a small positive association (*r* = 0.12) between first or second therapy session ratings of psychological treatment credibility and better mental health outcomes (Constantino et al., 2018a). The patients came from 24 cohorts, varying in size from 21 to 165. The interventions were typically forms of cognitive behavior therapy (individual or group). Outcomes were mainly anxiety or depression. A similar size of association (*r* = 0.15) was found in a meta-analysis with two thousand patients who primarily had diagnoses of anxiety or depression (Kumpasoğlu et al., 2025). Credibility was measured in all but one study with the Credibility/Expectancy Questionnaire (CEQ) (Devilly and Borkovec, 2000). A meta-analysis with almost thirteen thousand patients found a similar small association (*r* = 0.18) between positive initial expectations and better mental health outcomes (Constantino et al., 2018b). In this paper we set out to examine treatment credibility and expectancy in patients diagnosed with non-affective psychosis. In particular, we focused on patients with severe paranoia (persecutory delusions) who might be thought to be especially wary of interventions.

The meta-analyses were from studies of therapies delivered face to face. However, an individual participant data meta-analysis indicated that initial views of therapy may affect online therapies to a similar extent (Pontén et al., 2024). Subsequently, Dear (2025) examined data from four clinical trials involving eight hundred patients receiving internet-delivered treatments, either supported or self-guided, for depression, generalised anxiety, social anxiety, and panic disorder. Average scores for credibility (mean = 19.0) and expectancy (mean = 16.3) on the Credibility/Expectancy Questionnaire were high. There were no significant differences in scores whether the intervention was clinician-supported or self-guided. Being male, having had previous mental health treatment, and having lower anxiety levels were all associated with lower credibility and expectancy scores. Age was not associated with CEQ scores. Higher treatment credibility and expectancy predicted higher treatment uptake and improvements across all four symptom domains.

Treatment credibility and expectancy have received much less attention in the case of treatments for psychosis. Gaudiano and colleagues reported on a randomised controlled trial involving 46 inpatients with psychosis (Gaudiano et al., 2023). The trial compared Acceptance and Commitment Therapy to a time-matched supportive condition. High levels of credibility and expectancy were found for both allocation groups. Similarly, initial credibility and expectancy scores were high for 17 people hearing voices who received a brief coping intervention that included an app (Bell et al., 2020). A total score from the Credibility/Expectancy Questionnaire did not significantly predict end of treatment outcomes for paranoia, *r* = 0.00, or hallucinations, *r* = 0.07, for patients receiving an eight-week cognitive-behavioural internet intervention (Lüdtke et al., 2021). The internal reliability of the CEQ total score in this study was low (Cronbach's alpha = 0.6), indicating a potential measurement difficulty. Furthermore, the internet intervention had not been found in the intention-to-treat analysis of the clinical trial to lead to a significant between-group reduction in the measure of paranoia (Westermann et al., 2020), meaning that there may only have been a limited treatment effect to predict for paranoia. In a study of 36 people with psychosis admitted to a partial hospital programme providing pharmacological and cognitive-behavioural treatment, treatment expectancy ratings at admission did not predict outcomes at discharge on a composite symptom scale (Webb et al., 2013). In the same study, treatment expectancy did predict improvement in 420 patients with depression, with the authors concluding that treatment expectancies may play less of a role in psychosis. The limitation acknowledged by the study authors was the small size of the psychosis group, which means that the test of prediction would have been underpowered.

In this study we set out to examine treatment credibility and expectancy in a reasonably large group of patients with non-affective psychosis (schizophrenia, schizo-affective disorder, delusional disorder, psychosis not otherwise specified) receiving psychological interventions for persecutory delusions. We combined data from two clinical trials in which everyone was allocated to a psychological intervention and CEQ data were collected at the first treatment session. The aims were threefold: to assess how optimistic patients with current psychotic experiences are about psychological interventions; to identify potential predictors of less optimism; and, primarily, to test whether initial treatment credibility and expectancy predict improvement in the main presenting psychotic experience.

## 2 Methods

### 2.1 Participants

Treatment credibility and expectancy data collected from the first therapy session were used from 195 patients with persecutory delusions in the context of non-affective psychosis taking part in two clinical trials. 119 patients (of 130 total participants) were from the Feeling Safe trial (Freeman, Emsley et al., 2021a) and 76 patients (of 80 total participants) were from the THRIVE trial (Freeman et al., 2023). The 15 patients who did not provide treatment credibility and expectancy data from the trials did not significantly differ in the number of treatment sessions attended (mean = 13.5, SD = 8.3) compared to people who did provide the data (mean number of sessions = 12.3, SD = 8.5, *n* = 195), *p* = .601. The patients who did not provide this data also did not significantly differ in their severity of persecutory delusions at six months (mean PSYRATS score = 11.8, SD = 8.7, *n* = 14) compared to people who did (mean PSYRATS score = 12.0, SD = 5.3, *n* = 179), *p* = .646. The inclusion criteria for Feeling Safe were: aged 16 years or above; persistent (at least three months) persecutory delusion (as defined by Freeman and Garety (2000), held with at least 60% conviction; primary diagnosis of non-affective psychosis from the referring clinical team. The inclusion criteria for THRIVE were: aged 16 years or older with persistent (at least 3 months) persecutory delusions (as defined by Freeman and Garety (2000)) held with at least 50% conviction, who reported feeling threatened when outside around other people, and who had been given a clinical diagnosis of non-affective psychosis.

### 2.2 Assessments

The severity of persecutory delusions was assessed by research assistants, blinded to group allocation, before randomisation to therapy and again six months later. Other assessments were also completed with a research assistant prior to randomisation. Treatment typically began a fortnight after the baseline assessment. Credibility and expectancy ratings were taken at the first therapy session.

*Credibility/Expectancy Questionnaire (CEQ)* (Devilly and Borkovec, 2000). The scale is made up of three items assessing treatment credibility and three items assessing expectancy (see Table 2). Items 4 and 6 are converted to 1–9 scales for questionnaire total scoring. Factor analysis typically confirms the scale comprises two highly correlated factors (e.g. Coste et al., 2020; Sezer Katar et al., 2025). The same wording of the items was used in both treatment trials. Internal reliability of the scale in the current study was good to excellent (credibility: Cronbach's alpha = 0.83 (*n* = 195), expectancy: Cronbach's alpha = 0.92 (*n* = 193)).

*Psychotic Symptoms Rating Scale* (PSYRATS) - Delusions (Haddock et al., 1999). The PSYRATS-Delusions is a six-item measure that assesses the conviction, preoccupation, distress, and disruption associated with the main persecutory delusion. Higher scores indicate greater severity.

*Revised Green* et al *Paranoid Thoughts Scale (R-GPTS)* (Freeman et al., 2021b). The R-GPTS comprises eight-item ideas of reference scale and a 10-item ideas of persecution scale. Each item is rated on a 5-point scale. Higher scores indicate greater levels of paranoia.

*Columbia-Suicide Severity Rating Scale (C-SSRS)* (Posner et al., 2011). This is an interviewer-rated measure assessing suicidal ideation in the last month. The first item was used: the severity of the highest level of suicidal ideation experienced by the patient rated on an ordinal 6-point scale from 0 (none) to 5 (suicidal intent with plan).

*Warwick-Edinburgh Mental Well-being Scale (WEMWBS)* (Tennant et al., 2007). The WEMWBS is a fourteen-item scale assessing well-being over the past fortnight. Each item is rated on a 1 (none of the time) to 5 (all of the time) scale. Higher scores indicate greater well-being.

*Safety Behaviours Questionnaire—Persecutory Delusions* (SBQ) (Freeman et al., 2001). The SBQ is a semi-structured interview assessing the use of safety-seeking behaviours (actions that prevent the processing of disconfirmatory evidence) in the last month. The total number of avoidance behaviours was used.

*Time budget* (Jolley et al., 2006). The time budget measure assesses meaningful activity levels over the past week, completed during a structured interview, with four daily time blocks rated from 0 to 4. The rating scale is: 0 = nothing, 1 = predominantly passive activity, 2 = an independent activity requiring some planning and motivation, 3 = several 2-rated activities completely filling a time period or a more complex and demanding, but shorter, activity, 4 = time period filled with a variety of demanding independent activities. Higher scores indicate higher levels of meaningful activity.

EuroQoL (EQ-5D-5) (Herdman et al., 2011). The EQ-5D assesses health-related quality of life via questions about mobility, self-care, usual care, pain, and anxiety or depression. The EQ-5D index value score was used, with higher scores indicating better health. The Health Today index was also used, with individuals rating their health on a scale from 0 (worst health they can imagine) to 100 (best health they can imagine).

### 2.3 The treatments

In the Feeling Safe trial, participants were randomised to receive over six months either a weekly theory-driven psychological therapy (Feeling Safe) or an alternative weekly psychological intervention (befriending) from the same therapists.

Feeling Safe aims to help patients feel safer, happier, and able to get back to activities. In our theoretical model, persecutory delusions are conceptualised as inaccurate threat beliefs developed in the context of genetic and environmental risk. They are maintained by a number of psychological processes including excessive worry, low self-confidence, poor sleep, anomalous experiences, and safety-seeking behaviours (Freeman et al., 2025). The clinical implication of the model is that safety must be relearned. This is done by entering feared situations after the influence of the maintenance factors has been reduced. We have recently described the design principles, which are perceptible characteristics of the programme (Freeman, 2024). Feeling Safe is modular, personalised, and includes patient preference. After an initial assessment, participants are offered a tailored set of modules targeting up to five different areas (worry, self-confidence, the influence of voices, sleep, and re-learning safety while dropping safety-seeking behaviours). Modules are completed one at a time. The therapy was delivered by clinical psychologists in an average of 19 sessions.

Befriending, called ‘Feeling Safe and Supported’, followed a protocol that has previously been used successfully in two large clinical trials for patients with psychosis (Sensky et al., 2000; Jackson et al., 2008). The aim was to simulate regular interaction with a good friend: a general focus on non-threatening topics (although patients are not actively dissuaded from talking about concerns); non-confrontation; empathy; and supportiveness. We expected this intervention to provide a break for patients from their fears and encourage re-engagement in activities. The rationale provided to patients was: “The goal of Feeling Safe and Supported is to help you feel safer, happier, and doing more of what you want in life. We know that regular time to connect with other people is good for everyone's well-being. You will have regular time being listened to, respected, and talking about everyday topics. This takes our minds off difficulties and helps us to feel better about ourselves. In Feeling Safe and Supported, you will have time to reflect on interests and activities that you enjoy, which can help to increase motivation to do these activities and spark new interests. This all helps us to feel secure, calm, and connected.” The intervention was delivered by clinical psychologists in an average of 16 sessions.

In the THRIVE trial, participants were randomised to receive four sessions over a month of either a supported virtual reality (VR) cognitive therapy or a VR relaxation programme.

The treatment aim for the VR cognitive therapy (VRCT) was for patients to test their fear expectations regarding other people in order to relearn safety. VRCT comprised repeated behavioural experiments in VR to help patients learn that they were safer than they thought. The VR treatment was set in a (virtual) shopping centre. A virtual coach guided the person through the treatment. The coach encouraged the dropping of defence behaviours and elicited feedback to tailor the progression of the treatment. At the beginning of the first session, the person met the virtual coach in an office in the shopping centre. The coach explained the rationale behind the treatment. The participant then selected one of four virtual reality scenarios in which to begin. The scenarios were: a lift, a central atrium area, a café, and a clothes shop. Each offered five levels of difficulty (e.g. the number and proximity of people in the social situation increased) and participants worked their way through each level. To clarify the purpose of the treatment for patients, it was described as ‘VR Confidence Building’: we wanted people to feel more confident being in everyday situations around other people. The staff member, either a clinical psychologist or assistant psychologist, set up the virtual reality equipment, responded to questions, and at the end of the session helped the participant plan real-world tests to conduct between sessions. The average number of sessions attended was 3.7 (SD = 0.7).

In VR Mental Relaxation (VRMR) the staff member explained to patients that the way to deal with a fear about other people is to be calm in your own mind. Participants learned that VR mental relaxation achieves this by helping switch off our alarm systems, resulting in less anxiety and creating a sense of safety. VRMR would help patients become better at using mental relaxation techniques when they felt anxious. In each session, participants chose from a selection of calm VR environments (e.g. beach, forest, lake) in which to practise these techniques. The environments were non-social and taken from a commercially available VR relaxation programme. Every session patients completed two relaxation exercises, each lasting approximately 10 min. The exercises were played via an audio file. The staff member encouraged the patient to select a different VR environment for each exercise. Patients completed the exercises while seated and were offered a break between the two. At the end of the session, patients were encouraged to reflect on which exercises they found useful and to schedule time each day to practise them, both at home and outside in anxiety-provoking situations. The staff member (a clinical psychologist or assistant psychologist) provided the rationale for the therapy, set up the VR equipment, helped patients choose the environments, played the audio files, reviewed the exercises with participants, and assisted with homework setting (including making a check-in call or text during the week). The average number of sessions attended was 3.6 (SD = 1.0).

### 2.4 Analysis

SPSS Statistics Version 30.0 (IBM, 2024) was used. Descriptive statistics were used to summarise scores for credibility and expectancy by each of the two trials. Prediction of credibility and expectancy scores were assessed by Pearson correlation coefficients and then linear regressions controlling for therapy type. The predictive ability of credibility and expectancy for six-month persecutory delusion severity scores was assessed by linear regressions controlling for therapy type and baseline delusion severity. There was little missing data for the CEQ scale: one person did not complete one item and another omitted two items. These items were not prorated.

## 3 Results

### 3.1 The trial patients

Table 1 summarises participants' basic socio-demographic and clinical information. As is very typical of clinical trials of patients with current psychotic experiences, the average age was approximately 40, there were a greater number of men than women, and most participants were unemployed. The severity of the persecutory delusions was slightly greater in the Feeling Safe trial, likely the result of the higher delusion conviction entry criterion.

**Table 1:** Basic socio-demographic and clinical information.

Variable	Feeling Safe trial	VR trial
Mean age in years (SD)	41.1 (11.6)	39.7 (13.3)
Gender (n):		
Male	71 (59.7%)	46 (60.5%)
Female	48 (40.3%)	30 (39.5%)
Ethnicity (n):		
White	101 (84.9%)	61 (80.3%)
Black Caribbean	6 (5.0%)	3 (3.9%)
Black African	2 (1.7%)	2 (2.6%)
Black Other	1 (0.8%)	1 (1.3%)
Indian	3 (2.5%)	1 (1.3%)
Pakistani	3 (2.5%)	2 (2.6%)
Chinese	1 (0.8%)	1 (1.3%)
Other	2 (1.7%)	5 (6.6%)
Employment (n):		
Unemployed	93 (78.2%)	64 (84.2%)
Employed full-time	6 (5.0%)	1 (1.3%)
Employed part-time	9 (7.6%)	4 (5.3%)
Self-employed	1 (0.8%)	2 (2.6%)
Retired	2 (1.7%)	4 (5.3%)
Student	4 (3.4%)	0 (0%)
Housewife/husband	4 (3.4%)	1 (1.3%)
Diagnosis (n):		
Schizophrenia	73 (61.3%)	34 (44.7%)
Schizo-affective disorder	21 (17.6%)	13 (17.1%)
Delusional disorder	3 (2.5%)	5 (6.6%)
Psychosis NOS	22 (18.5%)	24 (31.6%)
Delusion severity at baseline:		
Mean PSYRATS score (SD)	18.2 (2.4)	16.9 (3.0)

### 3.2 Levels of credibility and expectancy

Table 2 presents mean scores on the CEQ. Scores are generally high across both trials. Both credibility, t(193) = 3.59, *p* < .001, and expectancy, t(191) = 3.64, p < .001, were significantly higher in the Feeling Safe trial than in the THRIVE trial. Credibility, t(117) = 1.39, *p* = .168, and expectancy, t(115) = 1.75, *p* = .083, did not significantly differ between Feeling Safe and befriending. Similarly, credibility, t(74) = 1.45, *p* = .152, and expectancy, t(74) = 0.72, *p* = .471, did not significantly differ between VR cognitive therapy and VR mental relaxation. Within the THRIVE trial, there were no significant differences between patients seen by clinical psychologists or assistant psychologists for credibility, t(74) = 0.78, *p* = .434, and expectancy, t(74) = 0.17, *p* = .865.

**Table 2:** Mean scores on the Credibility/Expectancy Questionnaire.

Trial	Therapy		Credibility	Expectancy
Feeling Safe	Feeling Safe	Mean (SD) n	21.2 (4.4) 58	19.6 (5.0) 58
	Befriending	Mean (SD) n	20.0 (5.0) 61	18.0 (5.0) 59
	Across trial	Mean (SD) n	20.6 (4.8) 119	18.8 (5.1) 117
THRIVE	VR Cognitive therapy	Mean (SD) n	18.8 (4.6) 39	16.4 (6.1) 39
	VR Mental Relaxation	Mean (SD) n	17.3 (4.7) 37	15.5 (4.8) 37
	Across Trial	Mean (SD) n	18.1 (4.7) 76	16.0 (5.5) 76
Across both trials		Mean (SD) n	19.6 (4.9) 195	17.7 (5.4) 193

Table 3 summarises patient scores on the individual items of the Credibility/Expectancy Questionnaire. A large majority of patients clearly anticipated benefit from the psychological interventions. For example, 92.5% of patients in the Feeling Safe trial, and 80.4% of patients in the THRIVE trial, thought that their treatment would be at least somewhat useful in helping them feel safer. 80.3% of patients in the Feeling Safe trial, and 69.7% of patients in THRIVE, felt that they would experience at least a 50% improvement. Only a small percentage of patients foresaw no benefit. Those who expected a 100% improvement were also only a small percentage.

**Table 3:** Endorsement rates for the Credibility/Expectancy Questionnaire items.

	Treatment credibility questions								
	*At this point, how logical does the therapy offered to you seem?*								
Trial	1 (not at all logical)	2	3	4	5 (somewhat logical)	6	7	8	9 (very logical)
Feeling Safe trial	0 (0%)	1 (0.8%)	3 (2.5%)	4 (3.4%)	25 (21.0%)	10 (8.4%)	20 (16.8%)	24 (20.2%)	32 (26.9%)
THRIVE trial	1 (1.3%)	0 (0%)	3 (3.9%)	1 (1.3%)	22 (28.9%)	11 (14.5%)	14 (18.4%)	10 (13.2%)	14 (18.4%)
	*At this point, how successful do you think this treatment will be in helping you feel safer?*								
	1 (not at all useful)	2	3	4	5 (somewhat useful)	6	7	8	9 (very useful)
Feeling Safe trial	0 (0%)	0 (0%)	8 (6.7%)	1 (0.8%)	29 (24.4%)	16 (13.4%)	30 (25.2%)	13 (10.9%)	22 (18.5%)
THRIVE trial	2 (2.6%)	0 (0%)	5 (6.6%)	8 (10.5%)	25 (32.9%)	10 (13.2%)	17 (22.4%)	4 (5.3%)	5 (6.6%)
	*How confident would you be in recommending this treatment to a friend who experiences similar problems?*								
	1 (not at all confident)	2	3	4	5 (somewhat confident)	6	7	8	9 (very confident)
Feeling Safe trial	2 (1.7%)	3 (2.5%)	4 (3.4%)	3 (2.5%)	15 (12.6%)	12 (10.1%)	19 (16.0%)	24 (20.2%)	37 (31.1%)
THRIVE trial	3 (3.9%)	2 (2.6%)	3 (3.9%)	6 (7.9%)	23 (30.3%)	3 (3.9%)	19 (25.0%)	8 (10.5%)	9 (11.8%)


	Treatment expectancy questions										
	*By the end of the therapy period, how much improvement in feeling safe do you think will occur?*										
	0%	10%	20%	30%	40%	50%	60%	70%	80%	90%	100%
Feeling Safe trial	1 (0.8%)	3 (2.5%)	4 (3.4%)	5 (4.2%)	10 (8.5%)	17 (14.4%)	13 (11.0%)	19 (16.1%)	22 (18.6%)	15 (12.7%)	9 (7.6%)
THRIVE trial	4 (5.3%)	0 (0%)	4 (5.3%)	6 (7.9%)	9 (11.8%)	14 (18.4%)	15 (19.7%)	8 (10.5%)	6 (7.9%)	7 (9.2%)	3 (3.9%)
	*At this point, how much do you feel that therapy will help you to improve your feelings of safety?*										
	1 (not at all)	2	3	4	5 (somewhat)		6	7	8	9 (very much)	
Feeling Safe trial	0 (0%)	6 (5.0%)	3 (2.5%)	10 (8.4%)	23 (19.3%)		14 (11.8%)	25 (21.0%)	19 (16.0%)	19 (16.0%)	
THRIVE trial	4 (5.3%)	2 (2.6%)	9 (11.8%)	7 (9.2%)	20 (26.3%)		14 (18.4%)	9 (11.8%)	5 (6.6%)	6 (7.9%)	
	*By the end of the therapy period, how much improvement in feeling safe do you really feel will occur?*										
	0%	10%	20%	30%	40%	50%	60%	70%	80%	90%	100%
Feeling Safe trial	0 (0%)	2 (1.7%)	6 (5.1%)	4 (3.4%)	11 (9.4)	16 (13.7%)	15 (12.8%)	24 (20.5%)	13 (11.1%)	19 (16.2%)	7 (6.0%)
THRIVE trial	4 (5.3%)	1 (1.3%)	4 (5.3%)	10 (13.2%)	4 (5.3%)	15 (19.7%)	16 (21.1%)	9 (11.8%)	6 (7.9%)	4 (5.3%)	3 (3.9%)

### 3.3 Associations with credibility and expectancy

Table 4 shows tests of associations between credibility and expectancy and the other variables. There were no significant predictors of treatment credibility score. Controlling for therapy type, higher levels of psychological well-being were associated with higher levels of expectancy. Higher levels of ideas of reference, but not persecutory ideation, were associated with higher levels of expectancy. There were no other significant associations with expectancy. Controlling for therapy type, gender did not significantly predict credibility, β = −0.09, *p* = .200, or expectancy, β = −0.04, *p* = .616. Controlling for therapy type, we found no indication that ethnicity (White ethnicity vs all others) predicted credibility, β = −0.08, *p* = .237, or expectancy, β = −0.02, *p* = .811.

**Table 4:** Associations of credibility and expectancy scores.

		Simple correlation		Controlling for therapy type (reporting standardised Beta)	
Variable		Credibility	Expectancy	Credibility	Expectancy
Credibility	Correlation		0.72⁎⁎⁎		0.70⁎⁎⁎
	*p*-Value		<0.001		<0.001
	n		193		193
PSYRATS total (delusion severity)	Correlation	0.04	0.03	−0.02	−0.034
	p-Value	0.612	0.688	0.742	0.632
	n	195	193	195	193
PSYRATS total at 6-months	Correlation	−0.18⁎	−0.18⁎	−0.19⁎	−0.18⁎
	p-Value	0.014	0.018	0.016	0.021
	n	179	177	179	177
R-GPTS Part A (ideas of reference)	Correlation	0.13	0.18⁎	0.10	0.16⁎
	p-Value	0.074	0.010	0.153	0.027
	n	194	193	194	193
R-GPTS Part B (persecutory ideation)	Correlation	0.09	0.101	0.04	0.05
	p-Value	0.190	0.162	0.630	0.533
	n	195	193	195	193
Columbia-Suicide Severity Rating Scale	Correlation	0.06	0.15⁎	−0.02	0.08
	p-Value	0.385	0.041	0.815	0.319
	n	189	187	189	187
Warwick-Edinburgh Mental Well-being Scale	Correlation	0.12	0.22⁎⁎	0.12	0.23⁎⁎
	p-Value	0.112	0.002	0.091	0.001
	n	192	190	192	190
Safety Behaviours Questionnaire—Avoidance	Correlation	0.10	0.15⁎	0.05	0.10
	p-Value	0.189	0.043	0.485	0.170
	n	191	189	191	189
Time Budget	Correlation	0.10	0.16⁎	0.07	0.13
	p-Value	0.188	0.035	0.346	0.067
	n	186	184	186	184
EuroQoL health index	Correlation	0.00	0.06	0.02	0.07
	p-Value	0.978	0.411	0.819	0.301
	n	190	188	190	190
EuroQoL health today	Correlation	0.06	0.14	0.04	0.12
	p-Value	0.390	0.061	0.545	0.083
	n	190	188	190	188
Age	Correlation	−0.07	−0.05	−0.08	−0.06
	p-Value	0.340	0.492	0.230	0.378
	n	195	193	195	193
Number of therapy sessions	Correlation	0.21⁎⁎	0.15⁎	0.00	−0.21
	p-Value	0.003	0.044	0.970	0.074
	n	195	193	195	193

⁎ *p* < .05.

⁎⁎ *p* < .01.

⁎⁎⁎ *p* < .001.

### 3.4 Outcomes at 6 months

Higher credibility scores at the start of therapy predicted lower delusion severity at six months (controlling for therapy type and baseline PSYRATS total score), standardised Beta = −0.17, *p* = .021. Higher expectancy scores at the start of therapy predicted lower delusion severity at six months (controlling for therapy type and baseline PSYRATS score), standardised = −0.17, *p* = .020. These were small associations explaining about 3% of the variance in outcomes.

## 4 Discussion

Our results for patients with severe paranoia in the context of a psychosis diagnosis mirror those found in meta-analyses for common mental health disorders such as anxiety and depression (Constantino et al., 2018a, Constantino et al., 2018b; Kumpasoğlu et al., 2025): initial perceptions of psychological treatment predict to a small degree later improvement in mental health symptoms. Even controlling for baseline severity of the persecutory delusions, we found a significant association between both treatment credibility and expectancy and persecutory delusion severity six months later. The caveat is that the variance explained in delusion outcome is small. Interestingly, the patients with severe paranoia typically reported high optimism about the treatments, albeit slightly lower for the shorter-term digital interventions than the lengthier face-to-face treatments. The levels of optimism were similar to previous studies with people with common mental health difficulties (e.g. Dear, 2025) and people with psychosis (e.g. Gaudiano et al., 2023; Bell et al., 2020). Patients with psychosis were generally optimistic about the potential of the psychological treatments. Those who were more pessimistic had lower levels of psychological well-being i.e. their confidence, cheerfulness, and optimism were generally low. However, overall there were very few predictors of initial perceptions of therapy. Severity of persecutory delusion, for example, had no significance. It will not be possible to gauge a person's views on the likely efficacy of their therapy unless they are asked. As such, we recommend routine assessment of treatment credibility and expectancy at the beginning of therapeutic work.

We would highlight the following strengths of this study. First, patients in the whole participant group received four different types of psychological treatments. As such, the results are not specific to one type. Second, assessments were conducted prior to the therapy session in which treatment perceptions were recorded, thus enabling tests of the prediction of credibility and expectancy. Third, follow-up assessments were conducted by blinded independent researchers, so accuracy will have been unaffected by assessor bias. However, the study also has important limitations. The data were collected in the context of clinical trials, leading to several biases. Therapists were largely clinical psychologists who were enthusiastic about the new treatments. Their optimism may have raised patient expectations. There are often several filters for patients entering a clinical trial (e.g. identification, referral, the informed consent process), so it is plausible that people less keen on psychological therapies would have been less likely to take part since there were many opportunities for clinicians or the person to decline participation. Views of treatments are unlikely to be static (Wester et al., 2024), meaning that capturing them at only one timepoint may underestimate their full significance. There will also be an important interaction between patient perceptions and an intervention's overall efficacy and performance in an individual instance. Clearly patient views of treatment would ideally reflect reality, albeit that appropriate optimism may help negotiate bumps in the road. It is also the case that patient responses to treatment follow individual trajectories. These trajectories may vary in their relationship to views of therapy (Jenner et al., 2024, Jenner et al., 2025). The potentially dynamic nature of treatment views, and variations according to trajectory, may mean that simple tests of associations within a group underestimate the importance of expectations for subgroups of patients. It is also likely that the measurement of credibility and expectancy could be improved. For instance, questions are worded towards positive views of intervention, which likely creates a bias. The greatest limitation of the study is that it tests associations, rather than causal relationships. If the factors that drive patient perceptions of credibility and expectancy can be discovered, these factors could be appropriately enhanced for patients and the potential benefits directly tested in a causal design.

## CRediT authorship contribution statement

**Daniel Freeman:** Writing – original draft, Methodology, Investigation, Funding acquisition, Formal analysis, Conceptualization. **Laina Rosebrock:** Writing – review & editing, Investigation. **Felicity Waite:** Writing – review & editing, Investigation.

## Funding

The Feeling Safe trial was funded by an 10.13039/501100000272NIHR Research Professorship awarded to Daniel Freeman (NIHR-RP-2014-05-003). The THRIVE trial was funded by the Medical Research Council Developmental Pathway Funding Scheme (MR/P02629X/1). Both trials were also supported by the NIHR Oxford Health Biomedical Research Centre (BRC). DF and FW are supported by the NIHR Oxford Health BRC. DF is an NIHR Senior Investigator. The views expressed are those of the authors and not necessarily those of the NIHR or the Department of Health and Social Care.

## Declaration of competing interest

None.

## References

[bb0005] Bell I.H., Rossell S.L., Farhall J., Hayward M., Lim M.H., Fielding-Smith S.F., Thomas N. (2020). Pilot randomised controlled trial of a brief coping-focused intervention for hearing voices blended with smartphone-based ecological momentary assessment and intervention (SAVVy): feasibility, acceptability and preliminary clinical outcomes. Schizophr. Res..

[bb0010] Constantino M.J., Coyne A.E., Boswell J.F., Iles B.R., Vîslă A. (2018). A meta-analysis of the association between patients’ early perception of treatment credibility and their posttreatment outcomes. Psychotherapy.

[bb0015] Constantino M.J., Vîslă A., Coyne A.E., Boswell J.F. (2018). A meta-analysis of the association between patients’ early treatment outcome expectation and their posttreatment outcomes. Psychotherapy.

[bb0020] Coste J., Tarquinio C., Rouquette A., Montel S., Pouchot J. (2020). Cross-cultural adaptation and validation of the French version of the credibility/expectancy questionnaire. Further insights into the measured concepts and their relationships. Psychol. Fr..

[bb0025] Dear B.F. (2025). Credibility and expectations: important factors for understanding clinical response, treatment completion, and dropout in internet-delivered psychological interventions. J. Consult. Clin. Psychol..

[bb0030] Devilly G.J., Borkovec T.D. (2000). Psychometric properties of the credibility/expectancy questionnaire. J. Behav. Ther. Exp. Psychiatry.

[bb0035] Freeman D. (2024). Developing psychological treatments for psychosis. Br. J. Psychiatry.

[bb0040] Freeman D., Garety P.A. (2000). Comments on the content of persecutory delusions: does the definition need clarification?. Br. J. Clin. Psychol..

[bb0045] Freeman D., Garety P.A., Kuipers E. (2001). Persecutory delusions: developing the understanding of belief maintenance and emotional distress. Psychol. Med..

[bb0050] Freeman D., Emsley R., Diamond R., Collett N., Bold E., Chadwick E., Isham L., Bird J., Edwards D., Kingdon D., Fitzpatrick R., Kabir T., Waite F., Oxford Cognitive Approaches to Psychosis Trial Study Group (2021). Comparison of a theoretically driven cognitive therapy (the Feeling Safe Programme) with befriending for the treatment of persistent persecutory delusions: a parallel, single-blind, randomised controlled trial. Lancet Psychiatry.

[bb0055] Freeman D., Loe B.S., Kingdon D., Startup H., Molodynski A., Rosebrock L., Brown P., Sheaves B., Waite F., Bird J.C. (2021). The revised Green et al., Paranoid Thoughts Scale (R-GPTS): psychometric properties, severity ranges, and clinical cut-offs. Psychol. Med..

[bb0060] Freeman D., Lister R., Waite F., Galal U., Yu L.M., Lambe S., Beckley A., Bold E., Jenner L., Diamond R., Kirkham M., Twivy E., CAusier C., Carr L., Saidel S., Day R., Beacco A., Rovira A., Ivins A., Nah R., Slater M., Clark D.M., Rosebrock L. (2023). Automated virtual reality cognitive therapy versus virtual reality mental relaxation therapy for the treatment of persistent persecutory delusions in patients with psychosis (THRIVE): a parallel-group, single-blind, randomised controlled trial in England with mediation analyses. Lancet Psychiatry.

[bb0065] Freeman D., Isham L., Waite F. (2025). A counterweight model for understanding and treating persecutory delusions. Psychol. Med..

[bb0070] Gaudiano B.A., Ellenberg S., Johnson J.E., Mueser K.T., Miller I.W. (2023). Effectiveness of acceptance and commitment therapy for inpatients with psychosis: implementation feasibility and acceptability from a pilot randomized controlled trial. Schizophr. Res..

[bb0075] Haddock G., McCarron J., Tarrier N., Faragher F.B. (1999). Scales to measure dimensions of hallucinations and delusions: the psychotic symptom rating scales (PSYRATS). Psychol. Med..

[bb0080] Herdman M., Gudex C., Lloyd A., Janssen M., Kind P., Parkin D., Bonsel G., Badia X. (2011). Development and preliminary testing of the new five-level version of EQ-5D (EQ-5D-5L). Qual. Life Res..

[bb0085] IBM Corp (2024).

[bb0090] Jackson H.J., McGorry P.D., Killackey E., Bendall S., Allott K., Dudgeon P., Gleeson J., Johnson T., Harrigan S. (2008). Acute-phase and 1-year follow-up results of a randomized controlled trial of CBT versus Befriending for first-episode psychosis: the ACE project. Psychol. Med..

[bb0095] Jenner L., Payne M., Waite F., Beckwith H., Diamond R., Isham L., Collett N., Emsley R., Freeman D. (2024). Theory driven psychological therapy for persecutory delusions: trajectories of patient outcomes. Psychol. Med..

[bb0100] Jenner L., Payne M., Waite F., Beckwith H., Diamond R., Isham L., Collett N., Emsley R., Freeman D. (2025). Learning how to improve the treatment of persecutory delusions: using a principal trajectories analysis to examine differential effects of two psychological interventions (Feeling Safe, Befriending) in distinct groups of patients. Schizophr. Bull..

[bb0105] Jolley S., Garety P.A., Ellett L., Kuipers E., Freeman D., Bebbington P.E., Fowler D.G., Dunn G. (2006). A validation of a new measure of activity in psychosis. Schizophr. Res..

[bb0110] Kumpasoğlu G.B., Campbell C., Saunders R., Fonagy P. (2025). Therapist and treatment credibility in treatment outcomes: a systematic review and meta-analysis of clients’ perceptions in individual and face-to-face psychotherapies. Psychother. Res..

[bb0115] Lüdtke T., Rüegg N., Moritz S., Berger T., Westermann S. (2021). Insight and the number of completed modules predict a reduction of positive symptoms in an Internet-based intervention for people with psychosis. Psychiatry Res..

[bb0120] Pontén M., Jonsjö M., Vadenmark V., Moberg E., Grannas D., Andersson G., Boersma K., Hedman-Lagerlöf E., Kleinstaeuber M., Weise C., Kaldo V., Ljótsson B., Andersson E., Axelsson E., Jensen K. (2024). Association between expectations and clinical outcomes in online v. face-to-face therapy–an individual participant data meta-analysis. Psychol. Med..

[bb0125] Posner K., Brown G., Stanley B., Brent D., Yershova K., Oquendo M., Currier G., Melvin G., Greenhill L., Shen S., Mann J. (2011). The Columbia-Suicide Severity Rating Scale. Am. J. Psychiatry.

[bb0130] Sensky T., Turkington D., Kingdon D., Scott J., Scott J.L., Siddle R., O’Carroll M., Barnes T.R.E. (2000). A randomized controlled trial of cognitive-behavioural therapy for persistent symptoms in schizophrenia resistant to medication. Arch. Gen. Psychiatry.

[bb0135] Sezer Katar K., Zengin İspir G., Danışman M. (2025). Psychometric properties of the credibility/expectancy questionnaire in individuals with opioid use disorder. J. Psychoactive Drugs.

[bb0140] Tennant R., Hiller L., Fishwick R., Platt S., Joseph S., Weich S., Parkinson J., Secker J., Stewart-Brown S. (2007). The Warwick-Edinburgh mental well-being scale (WEMWBS): development and UK validation. Health Qual. Life Outcomes.

[bb0145] Webb C.A., Kertz S.J., Bigda-Peyton J.S., Björgvinsson T. (2013). The role of pretreatment outcome expectancies and cognitive–behavioral skills in symptom improvement in an acute psychiatric setting. J. Affect. Disord..

[bb0150] Wester R.A., Schwartz B., Lutz W., Hall M., Hoos T., Rubel J. (2024). Treatment credibility as a mechanism of change in cognitive behavioral therapy: effects on depression and anxiety. J. Consult. Clin. Psychol..

[bb0155] Westermann S., Rüegg N., Lüdtke T., Moritz S., Berger T. (2020). Internet-based self-help for psychosis: findings from a randomized controlled trial. J. Consult. Clin. Psychol..

